# Variation of serum selenium concentrations in German sheep flocks and implications for herd health management consultancy

**DOI:** 10.1186/1751-0147-55-82

**Published:** 2013-11-19

**Authors:** Esther Humann-Ziehank, Philip C Tegtmeyer, Bjoern Seelig, Petra Roehrig, Martin Ganter

**Affiliations:** 1 Clinic for Swine, Small Ruminants, Forensic Medicine and Ambulatory Service, University of Veterinary Medicine Hannover, Foundation, Bischofsholer Damm 15, D-30173 Hannover, Germany; 2 Tieraerztliche Praxis Dr. Seelig, Krailing 5, D-65321 Heidenrod-Laufenselden, Germany

**Keywords:** Trace elements, Small ruminants, Mineral supplement, Pool samples, Selenium deficiency

## Abstract

**Background:**

This study was performed to demonstrate the widespread distribution and severity of selenium (Se) deficiency in sheep flocks and to evaluate the impact of influencing factors. In 150 flocks, ten serum samples of adult ewes were analysed for Se concentration. The farmers were interviewed concerning flock size, provision of mineral supplement, predominant form of husbandry (stationary fenced pasture/transhumance), predominant form of water provision (tap water/well/surface water) and predominant soil (sandy, silty/loamy, clay) in the area. The location of the flock was recorded as well as the production stage/season at the time of sampling. Intra-group variation and the validity to analyse pooled samples were tested.

**Results:**

Pools of five samples correlated well with the mean of individually analysed samples. The intra-group range of serum Se concentration varied enormously (mean 45.4 ± 18.8 μg Se/l). About 60% of the flocks showed mean serum Se concentrations below 80 μg/l, 37.4% were below 60 μg Se/l, representing a Se deficient stage. Using mineral supplement in general was no key factor for Se status. Stationary flocks on fenced pasture had constantly higher mean serum Se concentrations during breeding (outdoors, August-November), lambing (mainly indoors, December-March) and lactation (outdoors, April-July), whereas flocks practising transhumance had significantly lower Se status, except during lambing. There was no significant correlation between the soil type and the Se status, but flocks in Southern Germany tend to show a lower Se status compared to Central and Northern Germany. Increasing flock size was associated with lower mean serum Se concentrations. In stationary flocks only, the use of surface water was accompanied by significantly lower Se status.

**Conclusion:**

Se deficiency is widespread in German sheep flocks. More than one third of the flocks showed Se deficiency, indicating the need to optimise the nutritional management. Factors raising suspicion of Se deficiency are large flocks, transhumance during lactation and the breeding season as well as surface water provision in stationary flocks.

## Background

An adequate intake of essential trace elements is indispensable for animal health. The trace element selenium (Se) is known to be an important functional part of several selenoproteins. There are at least 25 different selenoproteins but the function of only a few is so far known [1]. The most investigated selenoproteins belong to the glutathione peroxidase (GPx) family, which has key functions in the anti-oxidative system. Whole blood GPx activity has been frequently used in ruminants to characterise the Se metabolism [2-4]. Nonetheless, one of our previously published experimental studies [5] underlined, that whole blood GPx reflects changes in the Se intake very slowly because of its dependence on newly formed erythrocytes restricting its diagnostic suitability. Recently, van Ryssen and colleagues [6] concluded that the serum Se concentration reliably reflects the current Se status of sheep. Moreover, serum Se analysis is a stable method, e.g. the inter-assay coefficient of variation being 6.2% for our laboratory. This facilitates comparisons between different studies. Clinical outcomes of Se deficiency are known and reported for sheep in many countries worldwide. However, the prevention strategies differ enormously due to country-specific markets and legislation. In Germany, ruminal boluses as well as vaccines or antiparasitic agents fortified with trace elements are not licensed for sheep. Among injectables, a combination of vitamin E and sodium selenite is on the market, but no long-term drugs. Oral mineral supplements (lick or powder) usually contain 20–60 mg sodium selenite/kg.

Overt and specific clinical manifestation of Se deficiency in sheep is nutritional muscular dystrophy occurring primarily in young lambs born to Se deficient dams. The range of non-specific disorders includes infertility, increased perinatal mortality, growth retardation and reduced disease resistance [7-9]. Germany was previously considered to provide low Se in regional grown feedstuff as published for cows [10] and sheep as well as for wild ruminants [11,12]. During the last decades, sheep production in Germany has decreased markedly and has been driven out to low quality pastures or has been used to contribute to landscape conservation (rural environment maintenance) resulting in mainly extensive husbandry of the herds. Whereas diagnosis of Se deficiency is very common in individual samples sent to our lab, comprehensive data at flock level including more than 2–3 samples per flock are currently missing. Therefore, Se status was studied as a part of a national project looking into several scientific issues including e.g. serological testing [13]. The results serve to demonstrate the widespread presence of Se deficiency in the nutritional management of sheep flocks and to evaluate influencing factors.

## Material and methods

In total, 150 German sheep flocks were sampled. All flocks were long-term clients of specialised veterinary sheep health consulting services provided by the authors. None of the flocks had a history of nutritional muscular dystrophy. Ten ewes per flock were chosen for sampling; selection criteria were the absence of distinctly impaired health status, age > 2 years and body condition score 3 or less. There was no selection for breed.

Venous blood was collected in individual serum tubes (S-Monovette, Sarstedt AG, Nümbrecht, Germany) without additives. Serum was obtained by centrifugation, transferred to another tube and deep frozen at −18°C until analysis. Serum Se concentration was analysed by graphite furnace atomic absorption spectroscopy (GFAAS, SOLAAR M, Thermo Fisher Scientific, Dreieich, Germany). The calibration was done using the standard addition method. Series of measurements were validated individually by using commercial controls at two Se levels (ClinChek® - Control, RECIPE GmbH, Munich, Germany). Additionally, an ovine control serum prepared by our laboratory was used for internal quality control. The mean inter-assay coefficient of variation was 6.2%, the detection limit 3 μg Se/l. Moreover, the method was certified annually by an external quality control programme offered by the Society for Advancement of Quality Assurance in Medical Laboratories (INSTAND e.V., Duesseldorf, Germany).

In 50 flocks, only results from pooled samples were available for evaluation. Two pools per flock were prepared by a thorough mixture of 5 × 0.1 ml of individual serum samples (use of Eppendorf Reference® Pipette, Scale 10-100 μl, Eppendorf, Hamburg, Germany). The pool samples were analysed as mentioned above. In 100 flocks, all ten samples were analysed individually. There is a controversy about the use of pooled samples in the literature. To prove the eligibility of integrating pooled sample results into our whole study evaluation, aliquots of 135 individually analysed serum samples from sheep were used additionally to produce 27 pools as described above. Pools as well as individual samples were analysed for Se concentration.

The classification of the Se status for the flocks as well as for individuals was assessed by the Se concentration as follows: > 80 μg Se/l = adequate; 60–79.9 μg Se/l = marginal; 30–59.9 μg Se/l = deficient; < 29.9 μg Se/l = severely deficient. Forage and pasture analysis was not feasible due to costs, efforts and frequent changing of pastures in some herds. Questionnaires were conducted by the authors on farms requesting the following data: Flock size (number of ewes), provision of commercial oral mineral supplement (yes/no), predominant form of husbandry (stationary fenced pasture or shepherding/transhumance), predominant form of water provision (tap water/ well/ surface water) and predominant soil (sandy soil, silty/loamy soil, clay soil) in the area. Moreover, the time of sampling was allocated to the sheep production stage/season: 1 = breeding period (outdoors, August-November), 2 = lambing period (mainly indoors, December-March) and 3 = lactation period (outdoors, April-July). The location of the flock was represented by the associated postal code. The flocks were categorised into flocks from Northern (n = 59), Central (n = 60) and Southern Germany (n = 28).

### Statistics

All statistical calculations were done using SAS 9.3. Data were checked for Gaussian distribution using the Shapiro-Wilk test. The results were given by mean and standard deviation (sd). Variability of Se concentration within flocks was assessed by calculating the total range (maximum – minimum Se concentration). The difference of two factors was tested using the Student t-test, higher numbers of factors were tested by analysis of variance and the Tukey’s Studentized Range (HSD) test. Analysis of linear regression was used to test congruence of pools vs. mean of individually analysed serum samples, mean Se status vs. flock size and mean Se status vs. range. To demonstrate the relative importance of the qualitative factors and the flock size regarding the dependent variables (mean Se concentration und range) we calculated the coefficient of determination using analysis of variance and linear regression analysis, respectively. The interaction of the qualitative variables with the flock size was tested by a one-way analysis of covariance and the F-Test for heterogeneity of the regression coefficients representing the interaction between two factors. The interaction of the qualitative factors was tested in pairs of two factors using a two-way-analysis of variance and the global F-Test for interaction.

All data analysed were collected as part of routine diagnosis (herd health management consultancy). Hence we did not seek ethical approval.

## Results

### Individually analysed samples compared to pooled samples

Regression analysis of serum Se concentration of pooled samples compared to mean of the same five samples analysed individually resulted in a highly significant linear regression line (y = 0.9713x - 1.271, R^2^ = 0.9745; p < 0.001; n = 135).

### General Se status of flocks and individuals

Classification of the flocks for Se status resulted in the following distribution: 42.5% = adequate Se status (> 80 μg Se/l), 20.0% = marginal Se status (60–79.9 μg Se/l), 30.8% = deficient Se status (30–59.9 μg Se/l) and 6.7% = severely deficient Se status (< 29.9 μg Se/l).

Regarding all samples analysed individually (100 flocks), the mean serum Se concentration was 64.4 (sd 31.8) μg Se/l, the lowest sample being 5.6 μg Se/l and the highest sample 159.1 μg Se/l. Classification of the individually analysed ovine samples resulted in the following distribution: 31.0% = adequate Se status, 18.4% = marginal Se status, 36.4% = deficient Se status and 14.3% = severely deficient Se status.

### Range of serum Se concentrations

The variation in the range of serum Se concentrations within the 10 samples per flock was very high. The mean range (difference between the lowest/minimum and highest/maximum sample) was 45.4 (sd 18.8) μg Se/l (n = 100 flocks). Regression analysis revealed a slight association of the Se status with the range (y = 0.3165x + 50.1, R^2^ = 0.0440, p = 0.04). Furthermore, the range was not associated with any other factor tested except the region: the mean range in Northern, Central and Southern Germany was 53.0 (sd 20.4) μg Se/l, 41.6 (sd 16.7) μg Se/l and 37.9 (sd 14.6) μg Se/l, respectively, the difference between Northern and Southern Germany being statistically significant (p < 0.05).

### Mineral supplements

Responses were given on the mineral supplement from 134 flocks. Nine flocks (6.7%) were offered no mineral supplement, whereas the majority (n = 125, 93.3%) were given an oral mineral supplement regularly. Mean serum Se concentrations were 54.1 μg Se/l (sd 18.8) and 73.6 μg Se/l (sd 32.4) in not-supplemented and supplemented flocks, respectively. The difference was not significant. The distribution to categories of Se status of the not-supplemented flocks was: adequate = one flock; marginal = three flocks; deficient = four flocks; severely deficient = one flock.

### Soil and region

Information on the regional soil composition was available from 132 flocks and allocated to the three predominant categories. The mean Se concentrations of the flocks were: Sandy soil: 71.8 μg Se/l (sd 28.7, n = 48), silty/loamy soil: 63.1 μg Se/l (sd 28.1, n = 29), clay soil: 74.5 μg Se/l (sd 28.4, n = 55). There was no statistical difference between the mean Se concentrations per flock regarding the soil type.

The flocks located in Northern and Central Germany revealed mean serum Se concentrations of 79.8 μg Se/l (sd 29.9, n = 59) and 71.9 μg Se/l (sd 25.7, n = 60), respectively. The mean of flocks in Southern Germany was 58.4 μg Se/l (sd 26.6, n = 28), the difference compared to flocks from Central and Northern Germany being significant (p < 0.05).

### Husbandry and production stage/season

**Figure 1:**
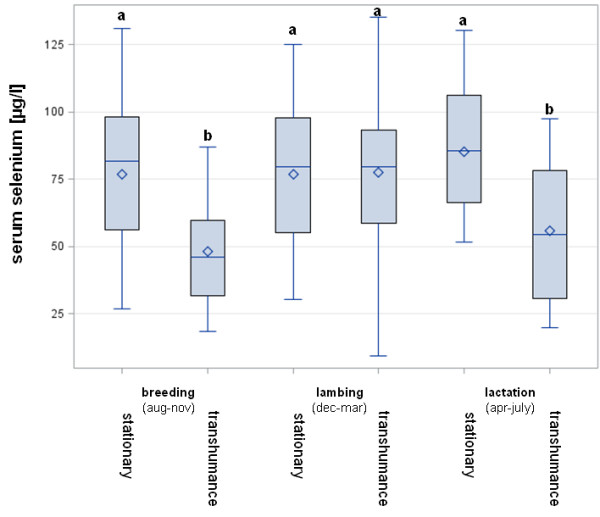
**Variation in the mean serum Se concentrations between flocks of different husbandry and production stage/season.** Whisker = min/max ; box = upper/lower quartile; line = median; ◊ = mean. Different letters indicate significant differences (p < 0.05).

The different forms of sheep husbandry revealed significant differences in the Se status. The flocks on stationary pastures showed a higher Se concentration (mean 78.8 μg Se/l, sd 26.2, n = 73) compared to flocks practising shepherding/transhumance (mean 61.1 μg Se/l, sd 28.0, n = 63; R^2^ = 0.0969, p < 0.001). The combined comparison of predominant form of husbandry and production stage/season is given in Figure 1. Whereas stationary flocks did not differ in mean Se concentrations comparing the three different production stages, the moving flocks (shepherding/transhumance) had significantly lower mean Se concentrations in the lactating and the breeding season, although there was no difference compared to the stationary flocks in the lambing period.

### Flock size

**Figure 2:**
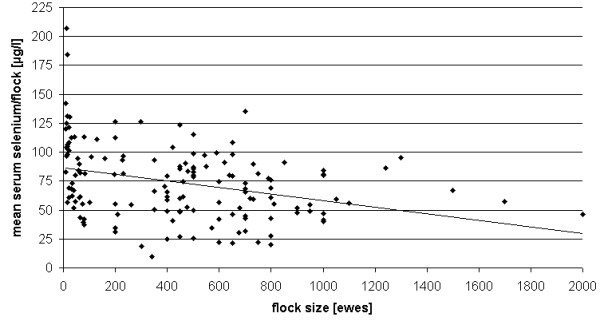
**Coherence of flock size and mean serum Se concentration.** (regression line: y = − 0.0284*x + 86.491; R^2^ = 0.1105; p < 0.0001; n = 150).

A larger flock size turned out to be slightly associated with lower mean serum Se concentrations as shown by a significant regression line (y = − 0.0284x + 86.491; R^2^ = 0.1105; p < 0.0001; n = 150; Figure 2). Generally, the mean flock size in stationary husbandry was lower (mean 286 ewes) compared to flocks practising shepherding/transhumance (mean 647 ewes).

### Water provision

**Figure 3:**
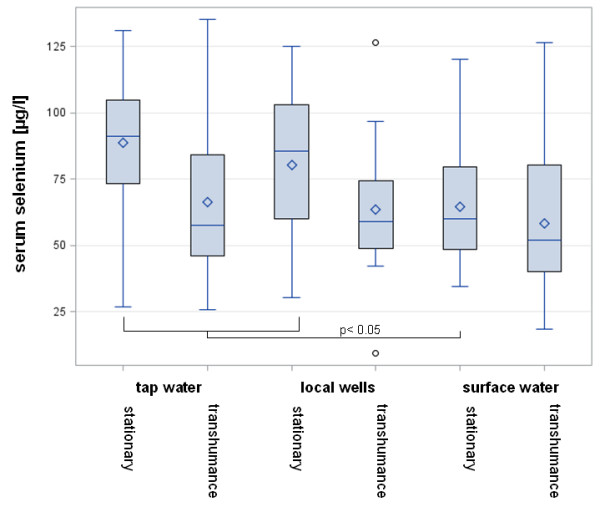
**Variation in the mean serum Se concentrations between flocks of different husbandry and water provision.** Whisker = min/max, box = upper/lower quartile, line = median; ◊ = mean; o = outlier.

Water provision was reported in 139 flocks: 40 flocks (28.8%) were provided with tap water, 44 flocks (31.7%) were provided with water from a local well and in 55 flocks (39.6%) surface water was used as the main water provision. The form of water provision was equally distributed to the two predominant forms of husbandry. In stationary fenced flocks the mean serum Se concentration was significantly lower when surface water was used (Figure 3).

### Relative importance and interaction of variables

The relative importance of variables was demonstrated by determining the individual coefficient of determination (R^2^) for each factor in terms of the dependent variables (mean Se concentration und range of Se concentration, respectively) as follows: Mineral supplement: R^2^ = 0.0228 and R^2^ = 0.0060; husbandry: R^2^ = 0.0969 and R^2^ = 0.0011; water provision: R^2^ = 0.0990 and R^2^ = 0.0111; soil: R^2^ = 0.0264 and R^2^ = 0.0266; region: R^2^ = 0.0699 and R^2^ = 0.1094; production stage/season: R^2^ = 0.0231 and R^2^ = 0.0069; The relative importance of the quantitative variable flock size in terms of the dependent variables was R^2^ = 0.1105 and R^2^ = 0.0132, respectively.

The test for interaction of variables regarding the mean Se concentration per flock resulted in two significant interactions only: Flock size*husbandry, p = 0.027; production stage/season* husbandry, p = 0.0029. There were no significant interactions of variables regarding the range of Se concentration.

## Discussion

The assessment of the nutritional status of farm animals is an important tool in herd health management. Adequate provision of essential trace elements is necessary to avoid nutritional maladies and to sustain animal production efficiency and animal welfare. During the last decades, Se deficiency has been noticed by veterinarians mainly in cases of clinical nutritional muscular dystrophy. The modern veterinary health services give greater attention to the Se status knowing that subclinical Se deficiency may have a detrimental effect on daily weight gain in lambs, reproduction in ewes and rams as well as full function of the immune system [8,9]. The number of samples sent to our lab for Se analysis in serum has increased markedly during the last 2–3 years and low Se concentrations are frequently being diagnosed. The presence of Se deficiency in Germany was previously assumed to be high in sheep and also in wild ruminants [11]. Nevertheless, background data from animals without clinical illness are not available. The data presented here are suitable for minimising this ‘gap in knowledge’.

The use of pool samples is an ongoing discussion in farm animal diagnostics [14,15]. As expected from the methodical point of view, our results showed that pooling of five samples correlated well with the mean of the same samples analysed individually for Se concentration. Nevertheless, the obvious disadvantage of pooling is the concealment of the possibly enormous range within the animals of one flock. The determined mean range of 45.4 μg Se/l at flock level was much higher compared to ranges in sheep fed a uniform amount of Se for scientific studies (mean range 23.4 μg Se/l, own unpublished observation). Knowledge of the range is essential for the management advice given, which may be to optimise equal access of all individuals to the mineral supplement. Therefore, the use of pools should be avoided in this instance.

The results underline that Se deficiency is very common in German sheep flocks. Whereas the use of the term ‘deficiency’ was for years restricted to cases with Se-related clinical dysfunctions, the Se supplementation needed for optimal health was characterised recently by full expression of Se-dependent selenoproteins in humans [16]. Our former study [5] demonstrated that there is a distinct increase of cytosolic GPx activity in all vital organs following a nutritional upgrade from 0.05 mg Se/kg dry matter (dm) to 0.2 mg Se/ kg dm in sheep. This was accompanied by increased serum Se concentrations from around 60 μg Se/l up to 90 μg Se/l followed by a plateau. This justifies a serum concentration of 80 μg Se/l to represent the benchmark for adequate Se supplementation in sheep as also published by others [17]. In the present study, 42.5% of the flocks only were classified as having an adequate Se status. The sheep in flocks with marginal Se status (20.0%) may compensate impaired selenoenzyme activity e.g. by other antioxidants as published for the clinical outcome of Se and/or vitamin E deficiency [12]. However, more than 37% of flocks investigated showed marked Se deficiency indicating the need to optimise the nutritional management. The Se status of the flocks was slightly positively associated with the range, but with a low coefficient of determination. However, if there is a generally low Se concentration in the total ration of the flock, the nutritional condition is the same for all individuals. In flocks showing a higher Se status the question of equal accessibility, e.g. to mineral supplements, might have a greater impact. These circumstances may partly explain the association between the Se status of the flocks and the range within the collected samples.

Some other factors tested seem to affect the mean serum Se concentration additionally. However, the relative importance of the individual factors was generally low.

Of course general provision of mineral supplements should be considered. In our study, determining the mean daily intake of Se per animal was not possible due to (i) the varying Se concentrations in the supplements, (ii) the diversity of chemical forms of Se in the supplements (sodium selenite, sodium selenate, organic forms etc.) and, in many cases, (iii) the lack of quantification of the mean daily intake in g/animal by the farmer. Analysing the Se concentration in the daily ration was not feasible due to costs. In the majority of flocks (93.3%) it was stated that mineral supplement was offered regularly. The difference between supplemented and not-supplemented flocks was not significant, which is probably caused partly by the numerical inequality (n = 125 vs. n = 9). However, the farmers’ statements regarding regular use of mineral supplements do not guarantee an adequate Se intake. This fact should be taken into account in the consulting process.

The assessment of the regional soil type was difficult to categorise due to frequent changes of pastures in some flocks. However, the three categories of soil types did not reflect differences in the mean Se status of the flocks. Nonetheless, geographically, there seemed to be a tendency of lower Se status in Southern Germany, which is in accordance with former studies on soil [18]. A relative importance of the factor ‘region’ on the dependent variable ‘range’ was indicated by our data (R^2^ = 0.1094), Southern Germany showing a significantly lower mean range compared to Northern Germany. Generally, forage Se concentration is largely determined by the prevailing geochemical and soil characteristics in the local environment; e.g. pH, redox conditions, speciation of Se, soil texture and mineralogy, organic matter content and the presence of competitive ions [19]. As it is impossible for the farmer/veterinarian to estimate the final forage Se concentration by environmental factors only, there is a need for validation at a forage or animal level. However, high variation in vegetation, e.g. in sheep used to contribute to landscape conservation, highly-frequent changes of pastures as well as high investigation costs render forage and pasture analysis unsuitable.

The predominant form of husbandry obviously caused differences in Se status with lower mean Se concentrations in flocks practising shepherding/transhumance. This finding is easy to explain, there being a logistic difficulty in feeding mineral supplements to moving flocks. Accordingly, the moving flocks differ significantly from stationary flocks during the outdoor season, although there was no difference from December to March during lambing time (Figure 1) which is an indoor time period in most of the German sheep flocks. The significant interaction between the production stage/season and the form of husbandry regarding the mean Se concentration/flock underlines the combined relevance of the factors.

There was a significant negative correlation between flock size and mean Se status of the herd (Figure 2), but with a low coefficient of determination (R^2^ = 0.1105) indicating the impact of additional factors. The significant interaction between the flock size and the form of husbandry regarding the mean Se concentration/flock may be explained by the fact that transhumance was mostly practised in larger flocks as mentioned above. Probably, the causal reason for this finding may be again the logistic challenge to allow equal access to mineral supplements for all animals in large flocks. Therefore, in large flocks the advice to check the Se status at animal level is required. Extra supplementation may be advisable e.g. during the breeding period, since the Se-dependent GPx is essential for e.g. embryo development [20] and sperm quality [21,22].

Moreover, there is a need for feasible concepts to provide Se supplementation for large and/or moving flocks which are not able to provide daily oral administration of mineral supplements. A viable strategy would be for example subcutaneous injection of sodium selenite (e.g. VitaminE-Selen-Loesung®, CP-Phama, Burgdorf, Germany) four weeks before breeding, four weeks before lambing as well as to newborn animals. As an alternative, the routine Se supplementation can be afforded by using a reticulorumin bolus [23,24] (not licensed in Germany) or Se enriched fertiliser [25].

The three alternative ways of water provision (tap water/ well/surface water) were equally represented in both forms of husbandry. The ‘water provision effect’ was predominant in the group on stationary fenced pasture when comparing tap and well water provision with surface water provision. In contrast, the group shepherding/transhumance showed mean serum Se concentrations of approximately 60 μg Se/l independently from the form of water provision. The amount of Se supplementation by water is irrelevant except in very special areas [26]. Therefore, this factor should be ineffective. However, the observed differences in stationary flocks may tentatively represent a completely different effect: The general care intensity! It is recommended and well-known in sheep production systems that surface water intake and wet areas should be avoided for sheep because of liver fluke and general endoparasite prophylaxis. Intensive effort on the part of the farmer is necessary to fence off access to surface water and to transport fresh water from wells or tap water pipes to the pastures by specials containers. One may speculate that increased care regarding water provision may be accompanied by increased care for adequate mineral supplementation.

## Conclusion

The widespread presence of subclinical Se deficiency in German sheep flocks was quantified by the presented data. The marked high intra-flock variation of serum Se concentration underlines the need for individual sample analysis, whereas pools cover relevant diagnostic information. More than one third of the flocks showed distinct Se deficiency, indicating the strong need to optimise the nutritional management. The farmers’ statements declaring regular administering of oral mineral supplements do not guarantee adequate Se status. The incidence of Se deficiency tends to be higher in Southern Germany. Influencing factors raising suspicion of Se deficiency are large flocks, shepherding/transhumance and outdoor seasons. Moreover, careless use of surface water in stationary fenced flocks may indicate low general care intensity including efforts administering mineral supplements. The Se status should be an issue of concern in herd health management consultancy and deficiencies should be eliminated to improve herd health and performance.

## Competing interests

The authors declare that they have no competing interests.

## Authors’ contributions

EHZ and MG conceived and designed the study. EHZ, PCT, BS and MG carried out the clinical trials, collected the blood samples and interviewed the farmers. PR prepared the serum pools and analysed the serum Se concentration. EHZ analysed the data and wrote the paper. All authors read and approved the final manuscript.
